# Applying the Information-Motivation-Behavioral Skills Model to a Virtual Reality–Assisted Cognitive Health Education Intervention to Promote Screening Uptake: Qualitative Study

**DOI:** 10.2196/91657

**Published:** 2026-10-21

**Authors:** Tingting Yin, Yulei Song, Bingqing Yang, Jingjing Yan, Yan Wang, Haiyan Yin, Xueqing Zhang, Guihua Xu, Yamei Bai

**Affiliations:** 1 School of Nursing Nanjing University of Chinese Medicine Nanjing, Jiangsu China

**Keywords:** mild cognitive impairment, dementia screening, virtual reality, IMB model, community nursing, health service navigation, qualitative research, information-motivation-behavioral skills model

## Abstract

**Background:**

Uptake of mild cognitive impairment (MCI) and dementia screening among community-dwelling older adults remains suboptimal globally and is shaped by limited dementia literacy, fear, stigma, and difficulties navigating services. Virtual reality (VR)–assisted education may enhance engagement, but older adults’ experiences of such education and the contextual conditions shaping screening intentions require further understanding.

**Objective:**

This study aims to explore community-dwelling older adults’ understanding, concerns, and attitudes regarding MCI and screening after exposure to a community-based VR-assisted cognitive health education intervention and to examine their perceived information, motivation, and behavioral skills related to screening using the information-motivation-behavioral skills (IMB) model.

**Methods:**

Twenty-two community-dwelling older adults were recruited from participating communities between August 23 and September 17, 2025, using purposive sampling with attention to variation in age and educational attainment, supplemented by snowball sampling. All participants completed a 40-minute VR-assisted cognitive health education session comprising a 10-minute immersive 360° video delivered through a Meta Quest 3 head-mounted display followed by 30 minutes of nurse-led face-to-face education. Four researchers coded the original Mandarin transcripts; themes and subthemes were derived inductively from the data and subsequently mapped to the IMB model.

**Results:**

Sixteen data-derived subthemes were subsequently mapped to the 3 domains of the IMB model. Information-related accounts encompassed misunderstanding and lack of knowledge about MCI, the relationship between MCI and dementia, knowledge acquisition and clarification, timing and psychological readiness, information overload, attention to peripheral details, and uncertain awareness of cognitive health status. Motivation-related accounts included fear and stigma associated with screening, perceived reduction in stigma and greater screening acceptability, reconciliation with aging, low self-prioritization, and a pessimistic outlook on treatment. Behavioral-skills accounts included low confidence in self-management, knowledge to action, community-based referral and navigation, and difficulty changing established habits. Across domains, participants’ accounts suggested that greater understanding and screening acceptability did not necessarily translate into action when practical navigation and referral support were lacking.

**Conclusions:**

Participants perceived VR-assisted cognitive health education as engaging and helpful for making MCI-related information more concrete, but their accounts also indicated that screening intentions may not translate into action without clear, trusted navigation and referral support. These findings describe participants’ experiences and perceived barriers and facilitators rather than estimating intervention effectiveness.

## Introduction

Dementia is a leading cause of disability and dependency in older adults and poses growing challenges for health systems worldwide as populations age [1,2]. In China, rapid demographic aging, large absolute numbers of older people, and uneven access to specialist services make cognitive impairment a major public health and nursing priority [3]. Mild cognitive impairment (MCI) represents an intermediate clinical state between normal aging and dementia, characterized by objective cognitive decline with relatively preserved functional independence, and it is widely regarded as an important window for early identification, risk reduction, and timely support [4,5]. Although disease-modifying treatment development has faced substantial setbacks and methodological challenges [6-8], prevention and risk reduction remain central components of dementia care [4,9]. Timely recognition may enable individuals and families to plan ahead, access services, and consider appropriate interventions and support [10]. However, the potential benefits of early detection depend on whether older adults are willing and able to engage with screening and follow-up services [10,11].

Despite increasing attention to cognitive health, dementia screening hesitancy remains common [11], and dementia may remain undetected in primary care [12]. Prior studies have identified multiple barriers, including limited dementia literacy [13,14], uncertainty about dementia risk reduction and assessment [13], fear of receiving a diagnosis and the psychological consequences of a high-risk result [15], and shame or anticipated stigma associated with dementia [15,16]. In addition, many older adults interpret memory lapses as a normal part of aging and therefore not warranting assessment [17]. These barriers involve not only information but also motivation, identity, family considerations, and social norms; system-level constraints and communication problems can further delay recognition and diagnosis [18].

Community-based primary care has a pivotal role in addressing these barriers. In China, community health centers are a core component of the primary health care system, with responsibilities for prevention and chronic disease management [19,20]. Primary care is also increasingly recognized as an important setting for early detection of cognitive impairment in older adults [21]. Strengthening coordination between primary care institutions and hospitals is a recognized priority in China [20], providing a structural basis for referral and navigation support when further cognitive assessment is needed.

Immersive virtual reality (VR) has been applied to cognitive assessment [22], dementia-related nursing interventions [23], and rehabilitation research [24], while systematic reviews have examined its health applications among community-dwelling older adults [25]. In dementia education specifically, a randomized trial in Japan evaluated a multielement VR program combining simulation and VR films with short films, lectures, and discussion; the intervention improved intention to help people with dementia, although the primary attitude outcome did not differ significantly between groups [26]. More recent work in older adults with MCI has also reported improvements in dementia knowledge, critical health literacy, and self-efficacy following a VR-based dementia prevention program [27]. These findings support the potential of VR as an educational format, but they do not explain how older adults experience VR-assisted cognitive health education in community settings or how such experiences intersect with screening decisions. The literature on immersive VR in community-dwelling older adults has also highlighted the need for further study of usability and acceptability [25]. In the Chinese context, qualitative evidence remains limited regarding how older adults perceive VR-assisted cognitive health education, how fear or stigma shape screening readiness, and what practical service-navigation barriers may persist after education. Clarifying these perceived processes and contextual conditions may help inform screening-promotion interventions that can be integrated with routine community care.

Behavior change theory provides a useful lens for understanding how educational interventions translate into health actions. The information-motivation-behavioral skills (IMB) model [28] proposes that people are more likely to adopt a health behavior when they (1) possess relevant and usable information, (2) are sufficiently motivated at personal and social levels, and (3) have the behavioral skills and self-efficacy to act, including the practical ability to navigate services. This model has been applied to a range of preventive and self-management behaviors [29-31] and is particularly relevant to screening contexts, where knowledge and willingness may be present but follow-through fails because the “next step” is unclear or difficult. Applying IMB to cognitive screening in community settings can clarify which components of an intervention are most salient for older adults and what additional nursing or system supports are needed to reduce the gap between intention and action.

Against this background, this study explored a community-based VR-assisted cognitive health education intervention. The aims of this qualitative study were to (1) describe community-dwelling older adults’ understanding, concerns, and attitudes regarding MCI and screening after exposure to VR-assisted education; (2) examine participants’ perceived information, motivation, and behavioral skills relevant to screening, guided by the IMB model; and (3) identify implications for community nursing practice and service processes that may support screening uptake.

## Methods

### Study Design

This study used a descriptive qualitative design to explore community-dwelling older adults’ experiences and perceptions of VR-assisted cognitive health education in relation to MCI screening. The qualitative component was initiated during the recruitment and health education phase of a broader community-based cluster-randomized study after the research team observed that some older adults were hesitant to participate in cognitive screening. It focused on participants’ accounts, meanings, and contextual factors and was not designed to test intervention effectiveness or estimate causal effects on screening uptake [32,33]. Reporting was guided by the COREQ (Consolidated Criteria for Reporting Qualitative Research) checklist [34] (Multimedia Appendix 1).

### Broader Cluster-Randomized Study and VR-Assisted Cognitive Health Education

The broader community-based cluster-randomized study comprised 3 study arms: VR-based adaptive-difficulty cognitive training, VR-based fixed-difficulty cognitive training, and a control condition without additional cognitive training. The community was the unit of randomization, and the 9 participating communities were allocated to the 3 study arms using a random-number-table method. A total of 178 older adults who met the parent-trial eligibility criteria, including confirmation of MCI, were formally enrolled in the 3 randomized trial groups. The shared VR-assisted cognitive health education component was provided across the study conditions, whereas the groups differed in the subsequent cognitive training component. The present qualitative sample was recruited earlier, during the common recruitment and health education phase, and participation in the qualitative interviews did not require a confirmed MCI diagnosis or depend on subsequent randomized-group allocation.

The shared VR-assisted cognitive health education session lasted approximately 40 minutes and comprised 2 components. First, participants viewed a 10-minute Chinese-language immersive 360° video using a Meta Quest 3 head-mounted display. Viewing was individual and seated. The experience was observational rather than interactive: participants could explore the 360° visual environment through head movements but did not manipulate virtual objects. The video presented first-person scenarios intended to depict cognitive change, including a familiar living room transforming into a floating island and the intermittent appearance of relatives’ faces in a “memory maze.” A local person living with dementia and a family member also described experiences of early screening, result notification, diagnosis, and treatment. Chinese subtitles were provided. Immediately after the VR session, participants received a 30-minute face-to-face educational session delivered by a nurse, who provided additional information about MCI and dementia and explained cognitive screening and the screening process.

Semistructured interviews were conducted with 22 older adults who had completed the shared VR-assisted cognitive health education session. The qualitative study specifically examined participants’ experiences of this common educational component and was not designed to compare experiences or outcomes across the randomized cognitive training groups. The interviews explored participants’ prior experiences of cognitive health education, reactions to the VR format, understanding and concerns regarding MCI and dementia, and views about screening and follow-through. The interview guide (Table 1) was informed by the IMB model and covered information-related understanding and misconceptions, motivational factors such as fear and stigma, and behavioral skills such as confidence and service navigation.

**Table 1:** Semistructured interview guide questions informed by the information-motivation-behavioral skills (IMB) model.

Number	Interview question	Core IMB constructs	Relevance to screening uptake or intervention experience
1	Before viewing the VR^a^ session, what did “MCI”^b^ and “dementia” mean to you? How did you interpret memory changes as “normal aging” versus “a problem”?	MCI or dementia literacy; symptom appraisal	Probing baseline misconceptions that shape perceived need for screening
2	Before today, what did you know about cognitive screening (what it involves, where it happens, and what results mean)?	Screening process knowledge; perceived consequences	Identifying knowledge gaps that create uncertainty and avoidance of screening
3	How do you usually experience community health talks or printed materials about cognitive health (eg, terminology, pace, and amount of information)?	Information accessibility; cognitive load	Examining barriers in conventional education that limit comprehension and engagement
4	After viewing the VR session, what information became clearer for you, and what (if anything) remained confusing?	Information acquisition; message clarity	Exploring whether participants perceived the VR session as clarifying information for decision-making
5	How acceptable was the VR format for you (eg, comfort, attention, and distraction by novelty or details)?	Information processing conditions; intervention acceptability	Clarifying delivery-related conditions that may strengthen or dilute key messages
6	When you think about cognitive screening, what feelings or concerns arise (eg, fear, being labeled, stigma, and “loss of face”)?	Stigma; anticipated social judgment; affective motivation	Exploring how stigma-related emotions may influence willingness to initiate screening
7	How do you view the value of screening and early action (eg, “knowing is useful” vs “nothing can be done,” treatment pessimism, fatalism)?	Outcome expectations; response efficacy; fatalism	Exploring how participants described the perceived value and manageability of screening and early action
8	Do responsibilities and self- prioritization influence your decision (eg, “not worth spending time or money on myself,” caregiving duties, and reconciliation with aging)?	Self-worth; aging narratives; competing priorities	Uncovering identity- and values-based barriers that persist despite improved knowledge
9	If you decided to be screened, what would you do first (where to go, which department, how to register), and what would be the hardest step?	Service navigation skills; self-efficacy	Mapping the intention-action gap into concrete capability and system-friction points
10	What support would make it easier to complete screening (eg, community referral, appointment help, reminders, and accompaniment)?	Social support; action planning; enabling resources	Identifying implementation supports participants considered helpful for translating readiness into screening action

^a^VR: virtual reality.

^b^MCI: mild cognitive impairment.

### Participants and Recruitment

Participants were recruited from 9 participating communities in Nanjing, Jiangsu Province, China, between August 23 and September 17, 2025, using purposive sampling supplemented by snowball sampling. Community staff posted standardized recruitment notices in community WeChat (Tencent Holdings Limited) group chats (WeChat is a widely used messaging and social media platform in China), including brief information about the study and the research team’s contact details. Interested older adults contacted the researchers directly and voluntarily. Among eligible individuals who expressed interest, the research team purposively selected participants with attention to variation in age and educational attainment to capture a range of perspectives. Snowball sampling was used as a supplementary strategy, whereby some participants learned about the study through older adults who had already participated. Eligibility criteria for the qualitative study differed from those of the parent trial because the qualitative study focused on experiences of cognitive health education and screening rather than cognitive-training outcomes among people with confirmed MCI. Eligibility criteria for the qualitative study were an age of 60-85 years, completion of the shared VR-assisted cognitive health education session, the ability to communicate sufficiently to participate in an in-depth interview, and willingness to discuss perceptions and experiences concerning MCI, dementia, and cognitive screening. A confirmed MCI diagnosis was not required. Individuals who had previously undergone MCI or other cognitive impairment screening at another institution and medical professionals were excluded. Participants provided written informed consent before the interview and received a small gift worth approximately CNY 10 (US $1.50) after participation.

### Data Collection and Analysis

Before each interview, the researcher collected participant characteristics including age, retirement status, marital status, educational level, family history of dementia, and prior MCI screening. The semistructured interview guide was developed in Mandarin Chinese and pilot-tested with 3 older adults, who were included in the final sample of 22. No substantive changes were made to the interview guide after pilot testing; therefore, the 3 pilot interviews were retained in the final analytic sample. During the pilot stage, interview notes were reviewed with the 3 pilot participants to confirm that the recorded content accurately reflected their intended meaning. Interviews were conducted in Mandarin by TY, a researcher trained in qualitative methods who was involved in volunteer recruitment and intervention delivery in the broader study. Before the qualitative interviews, TY had prior study-related contact with 7 of the 22 participants; she had no prior relationship with the remaining 15. All interviews were conducted face-to-face in private, quiet indoor rooms provided by the participating communities, with only the interviewer and participant present. Interviews lasted 39-152 minutes. The variation reflected differences in the depth and richness of participants’ responses; some participants provided particularly detailed and reflective accounts, leading to additional probing and clarification and consequently longer interviews. With participants’ consent, interviews were audio-recorded and transcribed verbatim in Mandarin within 24 hours after each interview. Field notes were recorded during and immediately after the interviews to document contextual observations, relevant nonverbal responses, and issues requiring clarification; these notes were considered alongside the transcripts during coding and interpretation. Thematic saturation was initially judged to have been reached after the 19th interview. Three additional interviews were conducted to confirm saturation, and no new themes emerged in interviews 20-22. Full verbatim transcripts from the 22 formal interviews were not returned to participants for checking.

Coding, theme development, and interpretation were conducted using the original Mandarin transcripts. All 4 researchers (TY, BY, JY, and YW) coded the full set of 22 transcripts using Microsoft Word and NVivo (version 15; Lumivero). Codes, subthemes, and themes were developed iteratively from the interview data. Coding decisions and emerging interpretations were compared regularly among the 4 researchers; disagreements regarding coding, code definitions, theme boundaries, or interpretation were discussed until consensus was reached. Related codes were grouped into candidate subthemes, which were reviewed against the coded extracts and original transcripts and refined until the research team agreed on the final thematic structure. After the subthemes had been developed from the data, they were mapped to the IMB model domains according to their predominant conceptual meaning. Thus, the IMB model served as a theoretical framework for organizing and interpreting the final thematic structure rather than as a rigid set of predetermined themes. English translations were prepared for the participant quotations selected for reporting after the qualitative analysis had been completed.

### Patient and Public Involvement

Patient and public involvement occurred at different stages of the broader research program and was separate from the 22 semistructured interviews reported in this study. First, formative qualitative work was conducted before this interview study to characterize community perceptions and stigma related to MCI and dementia; findings informed development of the VR-assisted cognitive health education content. During intervention development, community-dwelling older adults and community staff viewed the VR educational materials and provided feedback on the content and presentation. Their comments were considered by the research team when refining the materials before implementation. During recruitment for the present qualitative study, community health coordinators supported logistical organization and dissemination of recruitment information.

### Ethical Considerations

This study was approved by the Ethics Committee of the First Affiliated Hospital of Nanjing Medical University (number 2025-SR-015) and conducted in accordance with the Declaration of Helsinki [35]. Participants provided written informed consent before the semistructured interviews and received the stated compensation after participation.

## Results

### Characteristics of Participants

Twenty-two community-dwelling older adults from participating communities participated in the interviews. Their mean age was 73.27 (SD 4.95) years, and 15 (68.18%) were women. Most participants had completed secondary education or higher (Table 2). The relationship between the qualitative interview sample and the formally enrolled parent cluster-randomized trial sample is summarized in Table S1 in Multimedia Appendix 2. The two samples were recruited at different stages and under different eligibility criteria and were not statistically compared.

**Table 2:** Characteristics of participants in the semistructured interviews (N=22).

Demographic information			Value
Age (years), mean (SD)			73.27 (4.95)
**Sex, n (%)**			
	Male	7 (31.82)	
	Female	15 (68.18)	
**Marital status, n (%)**			
	Single	1 (4.54)	
	Married	20 (90.92)	
	Widowed	1 (4.54)	
**Education level, n (%)**			
	Primary or less	1 (4.54)	
	Middle school	8 (36.36)	
	High school	9 (40.92)	
	College and above	4 (18.18)	
**Retirement status, n (%)**			
	No	1 (4.54)	
	Yes	21 (95.45)	
**Family history of dementia, n (%)**			
	No	19 (86.36)	
	Yes	3 (13.64)	

### Themes, Subthemes, and Codes

Participants described varied prior understanding of MCI and dementia, concerns about screening, and experiences of the VR-assisted education session. Several participants perceived the VR content as making concepts easier to understand or less threatening, whereas others described information overload, distraction by delivery details, or persistent uncertainty. Across interviews, participants also emphasized practical barriers to acting on screening intentions, particularly uncertainty about where to go and how to obtain referral support. Sixteen subthemes were developed from the interview data and subsequently mapped to the 3 IMB domains (Figure 1).

**Figure 1:**
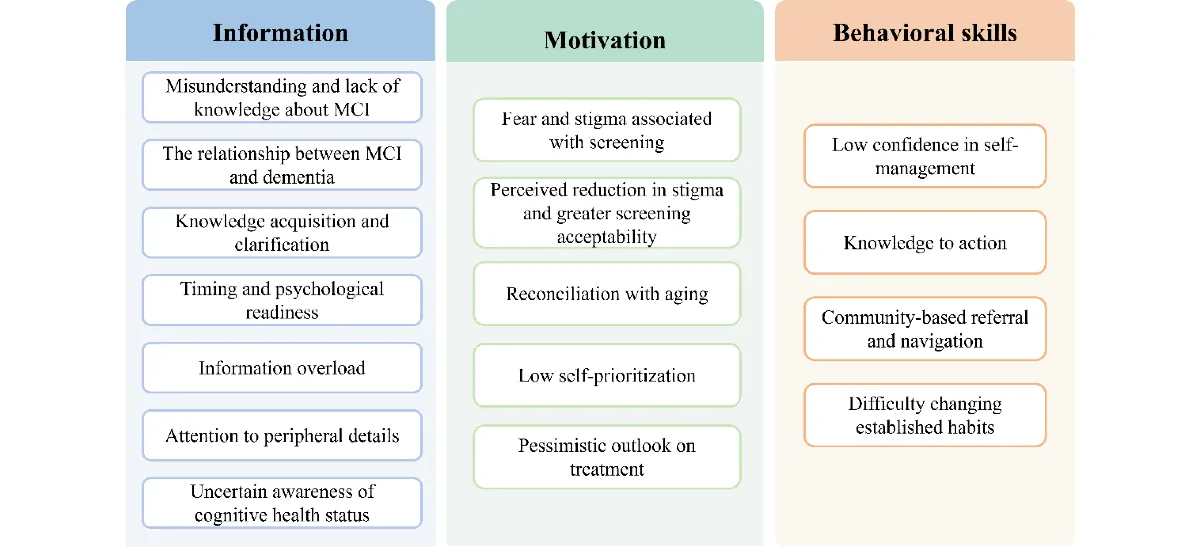
Subthemes mapped to the information-motivation-behavioral skills (IMB) model across 22 semistructured interviews. MCI: mild cognitive impairment.

### Information

#### Misunderstanding and Lack of Knowledge About MCI

Participants commonly described limited prior understanding of MCI, often equating memory problems with “dementia” or “mental problems.” Their accounts indicated that these misconceptions shaped how they interpreted the value and possible consequences of screening:

I always thought poor memory was senile dementia and there’s no cure for it, so I didn’t want to get screened.

N14, female, 68 years old

Participants’ accounts also reflected uncertainty about the nature of MCI and what, if anything, could be done after early identification. Some participants linked screening to additional worry or family burden:

In our village, they all say it’s the “confused disease,” and getting screened won’t help anyway; it just makes the children worried.

N21, female, 76 years old

#### The Relationship Between MCI and Dementia

Participants frequently described uncertainty about the distinction and relationship between MCI and dementia, and some interpreted MCI as inevitably progressing to dementia:

The VR said it’s not dementia, but it’s the early stage of dementia. So wouldn’t that just be a matter of time? Hearing this made me even more scared.

N12, male, 68 years old

Now I understand. It’s like the relationship between high blood pressure and cerebral infarction. If it’s managed early, it might not progress to that stage.

N15, male, 71 years old

#### Knowledge Acquisition and Clarification

Several participants perceived the visual and immersive presentation as easier to understand than professional terminology or dense written materials and described the session as helping them clarify previously confusing concepts about MCI and dementia:

Before, just listening to the terms, I couldn’t understand. The animation in the VR clearly demonstrated “aging of brain circuits” and “complete disconnection.” Now I really understand.

N5, male, 67 years old

Some participants also valued the individually delivered format and described it as allowing them to engage with the information at a more comfortable pace, without the social discomfort they associated with asking questions during face-to-face health education:

When the doctor was explaining, I was too embarrassed to ask. With this VR, I can watch it slowly and even go back to review it. Finally, I understood that “forgetfulness” and “pathological forgetfulness” are different.

N22, male, 71 years old

#### Timing and Psychological Readiness

Participants described timing and psychological readiness as important to how cognitive-health messages were received. When education occurred during periods of anxiety or poor physical health, some participants interpreted screening suggestions defensively or negatively:

During that time, I wasn’t feeling well physically either. Anyone who suggested I have my brain checked, I thought it was cursing me.

N4, female, 79 years old

Participants also described greater receptiveness at certain life transitions, such as retirement, suggesting that readiness to engage with prevention messages varied by personal context:

Before retirement, I never thought about these things. When I just retired, I was nervous. At this time, when someone told me about prevention, I could listen to them.

N20, female, 65 years old

#### Information Overload

Participants described conventional health-education materials and expert talks as difficult to follow when information was dense, highly technical, or delivered too quickly. These experiences were especially salient in accounts of frustration or disengagement:

With this VR, it feels like there’s a patient teacher guiding me step by step. Today, I’ll understand how the heart beats, and tomorrow I’ll learn about the blood vessels. This way of learning is not tiring, and I have a clear understanding. As a result, everything is firmly imprinted in my mind.

N8, female, 75 years old

Participants’ accounts suggested a preference for information delivered in smaller, layered segments rather than large amounts of technical content presented at once:

They gave me a booklet with densely packed text and many English abbreviations. After reading it for a while, I got a headache and threw it aside directly.

N1, female, 77 years old

The experts spoke too fast, with one professional term after another. I didn’t even have time to take notes and after listening, I became even more confused.

N10, male, 79 years old

#### Attention to Peripheral Details

Some participants reported that the novelty of VR or peripheral visual and operational details initially drew attention away from the core educational message. Questions about image accuracy or the realism of examples sometimes became a focus of discussion:

Why is the color of the brain model in the VR program pink? I heard it should be gray. Could this be unscientific?

N7, female, 69 years old

For these participants, attention to non–core details appeared to compete with attention to the intended message about MCI and screening:

The example given is that buying vegetables and making a mistake with the money. I think this example is not good. Our generation’s mental arithmetic is not as good as that of the young people.

N6, female, 79 years old

#### Uncertain Awareness of Cognitive Health Status

Participants often described uncertainty in appraising their own cognitive health, moving between interpretations of memory change as “normal aging” and as a possible problem. Some accounts combined worry with avoidance, including a preference not to know whether a problem was present:

I just occasionally forget things. Everyone does this. There’s no need to specifically check; finding out would only add to my worries.

N16, female, 75 years old

Sometimes I feel my reaction is slower, but I’m afraid it’s really a problem. It’s better not to know.

N13, female, 71 years old

### Motivation

#### Fear and Stigma Associated With Screening

Participants described screening as carrying negative social meanings, including being labeled, diagnosed, or viewed as “abnormal.” These anticipated consequences contributed to fear and reluctance in some accounts:

I don’t want to be screened. What if something goes wrong during the test, will others think I’m “abnormal”? Even my grandson might not let me do it.

N2, female, 71 years old

Some participants described avoiding cognitive screening because they feared discovering a problem or being judged by family, peers, or the wider community. Their accounts positioned stigma as a social and emotional barrier to screening:

I don’t go for this screening test even when my unit organizes it. I’m afraid it will affect my reputation and be said that “his mind is not good.”

N3, male, 82 years old

#### Perceived Reduction in Stigma and Greater Screening Acceptability

Some participants reported that the VR session made cognitive decline feel less shameful and screening more acceptable, particularly when the content framed cognitive change as a health issue that could be discussed and managed.

A man who had previously refused screening said after the session:

Previously, I thought being diagnosed meant being “mentally retarded.” Now I understand that it’s just a risk condition like high blood pressure, and early detection can also be controlled. I don’t fear being laughed at by others anymore.

N18, male, 81 years old

Participants who identified with the community-based cases described less embarrassment and greater openness to considering screening. These accounts suggest a perceived reduction in stigma rather than a measured change in screening behavior:

The cases in the VR were all normal. They were like neighbors to me, making me feel that having such a condition is not embarrassing. I should get checked if there’s a need.

N11, female, 69 years old

#### Reconciliation With Aging

Participants sometimes normalized cognitive decline as an inevitable part of aging. For some, this interpretation functioned as an adaptive way of maintaining calm and self-identity, but it also reduced the perceived need to seek assessment for memory changes:

As one gets older, the machine becomes old and the parts don’t work properly. This is normal. Why bother?

N3, male, 82 years old

Participants’ accounts indicated that acceptance of aging could coexist with reduced motivation to pursue screening, particularly when memory changes were interpreted as universal and unavoidable:

Having a poor memory also has its advantages. Not being able to remember the troubles, life becomes simpler.

N9, female, 73 years old

#### Low Self-Prioritization

Some participants placed their own health needs behind family responsibilities and described screening as not worth the time or expense. These accounts reflected low self-prioritization rather than lack of awareness alone.

A rural woman whispered:

It’s not easy for the children to earn money. I won’t bother to have my health checked. It’s a waste of money.

N1, female, 77 years old

Another man who had been caring for his ill spouse for a long time said:

My job is to take good care of my wife. I don’t care about my own situation.

N10, male, 79 years old

These accounts suggest that prioritizing family responsibilities over personal health could reduce motivation to seek screening.

#### Pessimistic Outlook on Treatment

Several participants expressed pessimistic expectations about the value of early identification or treatment for MCI and dementia. For some, the belief that progression was inevitable reduced the perceived benefit of screening:

I’ve seen what dementia is like in its advanced stage. Currently, there is simply no cure. Once diagnosed, there’s only waiting.

N20, female, 65 years old

Even with early intervention, it’s only a delay. Eventually, it will still progress. This awareness is actually a burden.

N14, female, 68 years old

### Behavioral Skills

#### Low Confidence in Self-Management

Some participants doubted that self-management or lifestyle changes could meaningfully protect cognitive health. These accounts reflected low self-efficacy and limited confidence in their ability to act on prevention advice:

Memory is determined by nature. What you eat and what you practice are all psychological effects.

N16, female, 75 years old

I can’t even manage my diabetes well. How can I manage my brain? Let it be as it is.

N19, female, 77 years old

#### Knowledge to Action

Participants described a gap between understanding screening recommendations and knowing how to act on them. Even when they considered screening worthwhile, some reported low confidence in making appointments, choosing a department, or initiating the process.

A retired teacher who was aware of the benefits of early intervention confessed:

I know I should get checked, but I’m completely confused about how to make an appointment, where to go, and what tests to take. It’s so confusing that I just put it off and forget about it eventually.

N9, female, 73 years old

Participants’ accounts repeatedly returned to the question of “what to do next,” highlighting the need for practical instructions, prompts, and support to initiate screening:

After the lecture, I thought it made sense, but when I got home and was busy with daily chores, I forgot all about it. There was no reminder, no one to guide me through the first step, and it was very difficult for me to start.

N21, female, 76 years old

#### Community-Based Referral and Navigation

Participants described familiar community health services as a preferred first point of contact for cognitive screening and referral. They emphasized the value of clear, convenient, and trusted guidance about where to go and how to access the appropriate service:

If the community hospital can conduct simple initial screenings, or if the doctor can refer me to the right department, I will definitely be willing to go. I myself would go to the hospital for registration, but I couldn’t even figure out whether to go to the neurology department or the geriatrics department.

N17, female, 69 years old

Participants’ accounts portrayed community staff and local networks as sources of practical guidance, trust, and reassurance. These relationships appeared important when older adults felt uncertain about navigating hospital departments or registration processes:

Our building’s group leader organized everyone to go to the community health center for assessment. With companions and familiar doctors, I felt at ease and I participated.

N12, male, 68 years old

#### Difficulty Changing Established Habits

Participants also described difficulty changing long-established lifestyle and social routines, even when they understood health recommendations. These accounts reflected challenges with self-regulation and translating advice into sustained behavior:

The doctor told me to go out more and play cards with others, but I’m used to staying at home alone. I don’t know who to go to when I go out. Thinking about it, it’s more comfortable to stay at home.

N3, male, 82 years old

I know eating lightly is good for the brain, but I’ve been eating salty food all my life and the dishes have lost their flavor. I just can’t eat them. My willpower is not strong enough.

N5, male, 67 years old

Participants’ accounts suggested that established habits could make both preventive lifestyle change and participation in screening activities more difficult. They described these routines as obstacles to acting on health advice and attending screening:

I usually enjoy going fishing and playing cards. These are all static activities. I just don’t like going out for activities, let alone participating in screening events in the community or at the hospital. I don’t like it at all and I can’t change that.

N5, male, 67 years old

## Discussion

### Principal Findings

This qualitative study explored how community-dwelling older adults experienced VR-assisted cognitive health education and how their accounts related to the IMB model. Participants described the immersive educational content as making some information about MCI and screening more concrete and understandable, and some reported feeling less fearful or embarrassed about cognitive screening after the session. However, participants also described persistent uncertainty about how to proceed with screening, including where to go, how to register, and whom to approach for assistance. Taken together, these accounts suggest potential information-related, motivation-related, and behavioral skill–related processes through which VR-assisted cognitive health education may support screening readiness, while also highlighting practical conditions that may prevent willingness from translating into action. These findings represent participants’ experiences and perceived processes and should not be interpreted as evidence that VR caused an increase in screening uptake.

### Information: Dementia Literacy and Cognitive Load

Participants described substantial gaps in basic knowledge about MCI, commonly conflating MCI with dementia or mental illness, and equating screening with being diagnosed and socially labeled. These accounts are consistent with studies documenting limited dementia knowledge in diverse community populations [13,14,36] and qualitative work showing that biomedical terminology, shame, and negative social meanings can shape how people understand dementia [16]. Help-seeking research further suggests that early cognitive changes are often discounted as normal aging, left uncertain, or misattributed, which can delay recognition of the need for assessment [17]. In our study, misconceptions were not only about disease concepts but also about the purpose and consequences of screening, with some participants anticipating social harm (loss of reputation) or family disruption (worrying adult children), which reduced perceived benefit.

Compared with their prior experiences of conventional health education, participants often described the VR session as easier to understand because it provided concrete visual explanations and could be followed at an individual pace. This perceived benefit is consistent with emerging intervention evidence showing that VR-based dementia education can support dementia-related knowledge, health literacy, or behavioral intentions in some settings [26,27]. In this study, however, participants’ descriptions of the immersive presentation should be interpreted as perceived clarity rather than evidence that knowledge objectively improved.

At the same time, participants described limitations related to novelty, peripheral details, and dense information. These concerns are consistent with systematic review evidence emphasizing the need to consider usability and acceptability when applying immersive VR with older adults [25]. Participants’ accounts in this study also suggested a preference for information that is paced and layered rather than densely presented. A practical implication is therefore to keep VR education brief and focused on a small set of core messages: what MCI is and is not, why screening may be considered, what screening involves, and what the next steps are in local services.

### Motivation: Stigma, Avoidance, Fatalism, and Reconciling With Aging

In the motivation domain, participants’ accounts highlighted fear of being labeled, anticipated discrimination, and concerns about face and reputation. This pattern is consistent with prior research identifying stigma, fear, and social consequences of a cognitive label as barriers to screening, diagnostic assessment, or dementia-service use [11,15,37]. Some participants described the community-based cases in the VR session as relatable and less stigmatizing, suggesting that familiar narratives may make screening feel more acceptable. Because these are retrospective qualitative accounts, however, the data do not establish the magnitude or durability of any change in stigma.

Several participants also expressed low perceived self-efficacy and fatalism, including skepticism that lifestyle changes or early identification could help. Pessimism about treatment and low self-prioritization were also evident in some accounts. These themes are consistent with prior research showing that reluctance toward cognitive screening or follow-up can be shaped by perceived limited benefits, psychological burden, stigma, and concerns about the consequences of diagnosis for patients and families [11,13,37]. Some participants described MCI as feeling more manageable after the session, particularly when it was framed as a risk state for which early management may still be meaningful; this should be interpreted as a perceived shift in understanding rather than a measured intervention effect.

A contribution of this study is the detailed description of reconciliation with aging as an adaptive psychological stance. Many participants normalized cognitive decline as part of aging and described acceptance as a way to maintain calm and identity. Similar narratives appear in help-seeking research, where memory changes are often discounted as normal aging or their significance is left uncertain [17]. In our data, this stance was double-edged: it protected well-being but reduced intrinsic motivation to pursue screening. Participants’ accounts suggest that the educational content did not necessarily require directly challenging acceptance of aging. For some participants, framing screening in relation to self-care, maintaining independence, and reducing potential family burden appeared to provide an alternative way of considering screening. For nursing practice, motivational work may therefore benefit from culturally sensitive and values-based communication rather than emphasizing disease threat alone.

Women accounted for 15 of the 22 interview participants. Although several female participants discussed health education in relation to family roles and caregiving, the study was not designed to compare women and men, and the overrepresentation of women may reflect self-selection into health education or interview participation. These accounts therefore should not be interpreted as evidence of a sex or gender difference in interest in the intervention.

### Behavioral Skills: The Intention-Action Gap and the Centrality of Community Referral

A practice-relevant pattern in the behavioral-skills domain was the gap participants described between considering screening worthwhile and knowing how to complete it. Participants frequently reported uncertainty about where to go, how to register, which department to choose, what screening would involve, and how to take the first step. In their accounts, this intention-action gap was closely tied to complex service navigation and low confidence in navigating hospital systems.

Consistent with research on primary care and community-based models of care, participants emphasized the value of a trusted and convenient referral route. Prior studies have highlighted the role of primary care in cognitive detection [21], the importance of coordination with specialist services in dementia diagnosis [38], and the value of integrated teams and navigation support for older adults [39,40]. In this sample, participants’ accounts suggest that education alone may be insufficient when there is no immediate, low-barrier next step; this is an implementation hypothesis that requires prospective evaluation.

For practice, the findings suggest a potential role for community nurses and primary care teams in linking education to action. Future VR-assisted education could be kept brief and structured, followed by facilitated discussion to check understanding and address concerns; multielement VR dementia education has previously combined immersive content with lectures and discussion [26]. It could also end with concrete service information: where screening is available, how to book, what to expect, and who can assist. Nurse-led navigation strategies such as appointment assistance, reminders, or accompaniment should be evaluated rather than assumed to improve uptake.

For education, nursing curricula and continuing professional development should strengthen skills in stigma-sensitive communication, motivational interviewing with older adults, and age-friendly digital health facilitation. Training should also cover how to recognize and respond to reconciliation-with-aging narratives in a way that respects values while supporting preventive action. For policy and service planning, investing in technology alone is unlikely to be sufficient. This emphasis is consistent with evidence supporting stronger coordination between primary care institutions and hospitals in China [20], navigation support for older adults using community-based primary care [39], and interdisciplinary team support in dementia primary care [40]. Future implementation strategies could evaluate standardized community-to-hospital referral processes, clear responsibility for follow-up, and protected staff time for navigation support. Program evaluation could consider not only knowledge-related outcomes but also screening completion and appropriate follow-up care.

### Strengths, Limitations, and Future Research

A strength of this study is its theory-informed qualitative design conducted within a community intervention context, which allowed exploration of experiences and contextual factors that quantitative outcomes may not capture. Developing themes from the interview data and subsequently mapping the subthemes to the IMB model provided a structured way to distinguish information, motivation, and behavioral-skill issues while retaining patterns grounded in participants’ accounts.

Several limitations should be considered. The sample was drawn from communities in one province and included many participants with secondary education or higher, which may limit transferability to rural or lower-literacy populations who could face different barriers to VR use and screening access. Participants initially volunteered in response to community recruitment information and were then purposively selected with attention to age and educational variation; this process may still have favored older adults who were more willing to engage with cognitive health education or research. Women were overrepresented (15/22), and interview participation may therefore have been subject to self-selection; apparent gender-related patterns should be interpreted cautiously. Future research should prospectively evaluate integrated approaches that combine VR-assisted education with structured nurse-led clarification, referral, reminders, and follow-up and should assess equity, feasibility, and screening completion in underserved settings.

### Conclusion

This qualitative study suggests that VR-assisted cognitive health education may be experienced as engaging and understandable by some community-dwelling older adults, while fear, stigma, uncertain self-appraisal, and practical service-navigation barriers can persist. Participants’ accounts particularly emphasized the value of trusted community-based guidance when considering what to do after receiving screening information. These findings support further evaluation of models that pair age-friendly digital education with clear referral and navigation support, but they do not establish that VR itself increases screening uptake.

## Appendix

Multimedia Appendix 1 COREQ checklist.

Multimedia Appendix 2 Relationship between the qualitative interview sample and the parent cluster-randomized trial sample.

## Abbreviations

COREQ: Consolidated Criteria for Reporting Qualitative Research
IMB: information-motivation-behavioral
MCI: mild cognitive impairment
VR: virtual reality

## Data Availability

The datasets used and analyzed during this study are available from the corresponding author on reasonable request.
