# Supplementary material for: Applying the Information-Motivation-Behavioral Skills Model to a Virtual Reality–Assisted Cognitive Health Education Intervention to Promote Screening Uptake: Qualitative Study
Source: JMIR Hum Factors. 2026 Oct 21;13:e91657. doi: 10.2196/91657 (PMC13735176; doi:10.2196/91657)
Supplement: Supplementary file 1 [file humanfactors_v13i1e91657_app1.docx]

Consolidated criteria for reporting qualitative studies (COREQ): 32-item checklist

| **No** | **Item** | **Guide questions/description** |
| --- | --- | --- |
| **Domain 1: Research team and reflexivity** | | |
| **Personal Characteristics** | | |
| 1. | Interviewer/facilitator | Tingting Yin |
| 2. | Credentials | Tingting Yin (PhD), Yulei Song (PhD), Bingqing Yang (M.S.), Jingjing Yan (M.S.), Yan Wang (M.S.), Haiyan Yin (PhD), Xueqing Zhang (M.S.), Guihua Xu (PhD), Yamei Bai (PhD) |
| 3. | Occupation | Tingting Yin (Student), Yulei Song (Expert in cognitive impairment research), Bingqing Yang (Student), Jingjing Yan (Student), Yan Wang (Student), Haiyan Yin (Expert in cognitive impairment research), Xueqing Zhang (Expert in cognitive impairment research), Guihua Xu (Professor of Nursing), Yamei Bai (Expert in cognitive impairment research) |
| 4. | Gender | female |
| 5. | Experience and training | All team members received training in qualitative research. The primary interviewer, Tingting Yin, had prior experience with qualitative interviewing. |
| **Relationship with participants** | | |
| 6. | Relationship established | Study-related familiarity existed with some participants before the qualitative interviews. |
| 7. | Participant knowledge of the interviewer | Before the qualitative interviews, Tingting Yin had prior study-related contact with 7 of the 22 participants through volunteer recruitment and intervention activities in the broader study; she had no prior relationship with the remaining 15 participants. |
| 8. | Interviewer characteristics | The primary interviewer was trained in qualitative methods and was involved in volunteer recruitment and intervention delivery in the broader community-based study. |
| **Domain 2: Study design** | | |
| **Theoretical framework** | | |
| 9. | Methodological orientation and Theory | Descriptive qualitative design with inductive thematic analysis; the final sub-themes were subsequently mapped to the Information–Motivation–Behavioral Skills (IMB) framework for organization and interpretation. |
| **Participant selection** | | |
| 10. | Sampling | Purposive sampling with attention to variation in age and educational attainment, supplemented by snowball sampling. |
| 11. | Method of approach | Community staff posted standardized recruitment notices in community WeChat group chats containing brief study information and the research team’s contact details. Interested older adults contacted the research team directly and voluntarily. Some potential participants also learned about the study through referrals from older adults who had already participated. |
| 12. | Sample size | 22 |
| 13. | Non-participation | No participant declined after being purposively selected and invited to complete the interview, according to the study records. |
| **Setting** | | |
| 14. | Setting of data collection | Face-to-face interviews were conducted in private, quiet indoor rooms provided by the participating communities. |
| 15. | Presence of non-participants | No |
| 16. | Description of sample | See Table 2 in the manuscript. |
| **Data collection** | | |
| 17. | Interview guide | An IMB-informed semi-structured interview guide was developed in Mandarin Chinese and pilot-tested with 3 older adults before formal data collection. The 3 pilot participants were included in the final sample of 22. The interview guide is presented in Table 1. |
| 18. | Repeat interviews | No repeat interviews were conducted. |
| 19. | Audio/visual recording | With participants’ consent, all interviews were audio-recorded and transcribed verbatim in Mandarin within 24 hours after each interview. |
| 20. | Field notes | Field notes were recorded during and immediately after interviews to document contextual observations, relevant nonverbal responses, and issues requiring clarification. Field notes were considered alongside the transcripts during coding and interpretation. |
| 21. | Duration | Interviews lasted 39–152 minutes. Variation reflected differences in the depth and richness of participants’ responses and the amount of probing and clarification required. |
| 22. | Data saturation | Thematic saturation was initially judged to have been reached after the 19th interview. Three additional interviews were conducted to confirm saturation; no new themes emerged in interviews 20–22. |
| 23. | Transcripts returned | No. Full verbatim transcripts from the 22 formal interviews were not returned to participants. During pilot testing, interview notes were reviewed with the 3 pilot participants to confirm that the recorded content accurately reflected their intended meaning. |
| **Domain 3: Analysis and findings** | | |
| **Data analysis** | | |
| 24. | Number of data coders | Four researchers (T.Y., B.Y., J.Y., and Y.W.) coded the full set of 22 Mandarin interview transcripts. |
| 25. | Description of the coding tree | Codes and sub-themes were developed iteratively from the interview data. Related codes were grouped into candidate sub-themes, which were refined against the coded extracts and original transcripts. The final sub-themes were subsequently mapped to the three IMB domains; the final thematic structure is presented in Figure 1. |
| 26. | Derivation of themes | Themes and sub-themes were derived inductively from the interview data and were subsequently organized within the IMB framework according to their predominant conceptual meaning. |
| 27. | Software | Microsoft Word and NVivo version 15 were used for data management, coding, organization, and analysis. |
| 28. | Participant checking | No. Participants did not review or provide feedback on the final themes or study findings. During pilot testing, however, the 3 pilot participants reviewed their interview notes to confirm the accuracy of the recorded content. |
| **Reporting** | | |
| 29. | Quotations presented | Yes. Participant quotations are presented to illustrate the themes and sub-themes. |
| 30. | Data and findings consistent | Yes. The reported findings are supported by participant quotations and are consistent with the thematic analysis. |
| 31. | Clarity of major themes | Yes. Major themes are clearly presented under the Information, Motivation, and Behavioral Skills domains. |
| 32. | Clarity of minor themes | Yes. Minor themes/sub-themes are clearly described and illustrated with quotations. |
