# Supplementary material for: Applying the Information-Motivation-Behavioral Skills Model to a Virtual Reality–Assisted Cognitive Health Education Intervention to Promote Screening Uptake: Qualitative Study
Source: JMIR Hum Factors. 2026 Oct 21;13:e91657. doi: 10.2196/91657 (PMC13735176; doi:10.2196/91657)
Supplement: Supplementary file 2 [file humanfactors_v13i1e91657_app2.docx]

**Multimedia Appendix 2**

Table S1. Relationship Between the Qualitative Interview Sample and the Parent Cluster-Randomized Trial Sample

| **Characteristic** | **Qualitative interview sample** | **Parent cluster-randomized trial sample** |
| --- | --- | --- |
| Sample size | 22 older adults | 178 older adults |
| Study stage | Recruitment and VR-assisted cognitive health education phase of the broader community-based study | Formal enrollment in the cluster-randomized trial after parent-trial eligibility assessment |
| Source setting | Participating communities | Nine participating communities |
| Primary purpose | To explore older adults’ experiences of VR-assisted cognitive health education and their perceptions of MCI screening | To evaluate different VR-based cognitive training strategies among eligible participants with MCI |
| MCI diagnosis required | No | Yes |
| Sampling/recruitment approach | Purposive sampling with attention to variation in age and educational attainment, supplemented by snowball sampling | Enrollment after meeting the predefined parent-trial eligibility criteria |
| Recruitment period relevant to the qualitative study | August 23–September 17, 2025 | Parent-trial recruitment period |
| Shared VR-assisted cognitive health education | Completed before the qualitative interview | Provided as the common cognitive health education component |
| VR health education component | 10-min immersive 360° VR video using a Meta Quest 3 head-mounted display, followed by 30-min nurse-led face-to-face education | Same shared VR-assisted cognitive health education component |
| Cognitive training allocation | Not used as an eligibility or sampling criterion for the qualitative study | Three cluster-randomized conditions: VR-based adaptive-difficulty cognitive training, VR-based fixed-difficulty cognitive training, and control without additional cognitive training |
| Unit of randomization | Not applicable to the qualitative study | Community |
| Qualitative interview | Yes; face-to-face semi-structured interview | Not a requirement of the parent trial |
| Main data analyzed in the present paper | Interview transcripts and field notes | Parent-trial outcome data are not analyzed in the present qualitative paper |

*Note: The qualitative interview sample and the parent trial sample were recruited at different stages and according to different eligibility criteria. In particular, a confirmed diagnosis of MCI was not required for participation in the qualitative study, whereas it was required for formal enrollment in the parent trial. The two samples are therefore presented for contextual clarification and should not be interpreted as directly comparable groups. No statistical comparisons between the two samples were performed. MCI, mild cognitive impairment; VR, virtual reality.*
